# The Global Brain Health Survey: Development of a Multi-Language Survey of Public Views on Brain Health

**DOI:** 10.3389/fpubh.2020.00387

**Published:** 2020-08-14

**Authors:** Isabelle Budin-Ljøsne, Barbara Bodorkos Friedman, Sana Suri, Cristina Solé-Padullés, Sandra Düzel, Christian A. Drevon, William F. C. Baaré, Athanasia Monika Mowinckel, Enikő Zsoldos, Kathrine Skak Madsen, Rebecca Bruu Carver, Paolo Ghisletta, Mari R. Arnesen, David Bartrés Faz, Andreas M. Brandmaier, Anders Martin Fjell, Aud Kvalbein, Richard N. Henson, Rogier A. Kievit, Laura Nawijn, Roland Pochet, Alfons Schnitzler, Kristine B. Walhovd, Larysa Zasiekina

**Affiliations:** ^1^Department of Genetics and Bioinformatics, Norwegian Institute of Public Health, Oslo, Norway; ^2^Department of Psychology, Center for Lifespan Changes in Brain and Cognition, University of Oslo, Oslo, Norway; ^3^Department of Psychiatry and Oxford Centre for Human Brain Activity, Wellcome Centre for Integrative Neuroimaging, University of Oxford, Oxford, United Kingdom; ^4^Department of Medicine, Facultat de Medicina i Ciències de la Salut, Universitat de Barcelona, Barcelona, Spain; ^5^Center for Lifespan Psychology, Max Planck Institute for Human Development, Berlin, Germany; ^6^Vitas AS, Oslo, Norway; ^7^Department of Nutrition, Faculty of Medicine, Institute of Basic Medical Sciences, University of Oslo, Oslo, Norway; ^8^Danish Research Centre for Magnetic Resonance, Centre for Functional and Diagnostic Imaging and Research, Copenhagen University Hospital Hvidovre, Hvidovre, Denmark; ^9^Department of Psychiatry and Wellcome Centre for Integrative Neuroimaging (WIN), University of Oxford, Oxford, United Kingdom; ^10^Communication Department, Norwegian Institute of Public Health, Oslo, Norway; ^11^Methodology and Data Analysis Unit, Faculty of Psychology and Educational Sciences, University of Geneva, Geneva, Switzerland; ^12^Faculty of Psychology, Swiss Distance University Institute, Brig, Switzerland; ^13^Swiss National Centre of Competence in Research LIVES, University of Geneva, Geneva, Switzerland; ^14^Norwegian Brain Council, Oslo, Norway; ^15^MRC Cognition and Brain Sciences Unit, Department of Psychiatry, University of Cambridge, Cambridge, United Kingdom; ^16^MRC Cognition and Brain Sciences Unit, University of Cambridge, Cambridge, United Kingdom; ^17^Department of Psychiatry, Amsterdam University Medical Centers, Amsterdam Neuroscience, Vrije Universiteit Amsterdam, Amsterdam, Netherlands; ^18^Belgian Brain Council, Université Libre de Bruxelles, Bruxelles, Belgium; ^19^Medical Faculty, Institute of Clinical Neuroscience and Medical Psychology, German Brain Council, Heinrich Heine University Düsseldorf, Düsseldorf, Germany; ^20^The Ukrainian Psychotrauma Center, Lesya Ukrainka Eastern European National University, Lutsk, Ukraine

**Keywords:** survey, brain health, perceptions, attitudes, lifestyle, cognitive health, mental health

## Abstract

**Background:** Brain health is a multi-faceted concept used to describe brain physiology, cognitive function, mental health and well-being. Diseases of the brain account for one third of the global burden of disease and are becoming more prevalent as populations age. Diet, social interaction as well as physical and cognitive activity are lifestyle factors that can potentially influence facets of brain health. Yet, there is limited knowledge about the population's awareness of brain health and willingness to change lifestyle to maintain a healthy brain. This paper introduces the Global Brain Health Survey protocol, designed to assess people's perceptions of brain health and factors influencing brain health.

**Methods:** The Global Brain Health Survey is an anonymous online questionnaire available in 14 languages to anyone above the age of 18 years. Questions focus on (1) willingness and motivation to maintain or improve brain health, (2) interest in learning more about individual brain health using standardized tests, and (3) interest in receiving individualized support to take care of own brain health. The survey questions were developed based on results from a qualitative interview study investigating brain health perceptions among participants in brain research studies. The survey includes 28 questions and takes 15–20 min to complete. Participants provide electronically informed consent prior to participation. The current survey wave was launched on June 4, 2019 and will close on August 31, 2020. We will provide descriptive statistics of samples distributions including analyses of differences as a function of age, gender, education, country of residence, and we will examine associations between items. The European Union funded Lifebrain project leads the survey in collaboration with national brain councils in Norway, Germany, and Belgium, Brain Foundations in the Netherlands and Sweden, the National University of Ostroh Academy and the Women's Brain Project.

**Discussion:** Results from this survey will provide new insights in peoples' views on brain health, in particular, the extent to which the adoption of positive behaviors can be encouraged. The results will contribute to the development of policy recommendations for supporting population brain health, including measures tailored to individual needs, knowledge, motivations and life situations.

## Introduction

Brain diseases represent a significant public health challenge (1). In Europe, 165 million people live with a brain disorder, amounting to a cost of 800 billion euros per year (2). Worldwide, neurological disorders such as stroke, epilepsy, multiple sclerosis, Parkinson's disease, brain tumors, and dementias are the second leading cause of death and the leading cause of disability (3). Mental health disorders including depression, anxiety, and schizophrenia affect about one third of the global population across the lifespan (4) and are associated with 10 years shorter life expectancy (5). Brain disorders negatively impact the quality of life and well-being of patients and their families and drain human and financial resources.

Brain health was recently proposed as an overarching concept to describe both measures to maintain a healthy brain and “health condition[s] related to function, diseases, injuries and disorders of the brain and other parts of the nervous system” (6). The American Heart Association and American Stroke Association define brain health as “an optimal capacity to function adaptively in the environment,” including “the ability to think, move, feel, pay attention, perceive, and recognize sensory input; to learn and remember, communicate, problem solve, and make decisions, to have mobility; and to regulate emotional status”(7). The U.S. National Institute on Aging supplements this definition by stating that “brain health is all about making the most of your brain and helping reduce some risks to it as you age” (8).

Research funders, research consortia, civil organizations, and governments progressively allocate resources to improve our understanding of brain function, prevent brain diseases, and strengthen the existing biomedical knowledge about brain disorders in health care systems. The European Commission has invested more than 6.3 billion euros in brain research since 2007 through its research and innovation framework programmes (2). Research projects for instance include Lifebrain (9), a research consortium exploring risk and protective factors influencing brain health across the lifespan, and the Human Brain Project (10), a research infrastructure in the fields of neuroscience and computing. Similarly, the European Brain Council (11) and its national counterparts raise awareness of the importance of brain health among European decision makers, support brain research, and work to improve the quality of life among individuals suffering from brain disorders (12).

Recent evidence shows that lifestyle behaviors and certain medical conditions impact cognitive function, mental health and dementia risk (13), thereby also influencing brain health. Physical activity has been consistently shown to be associated with reduced risk of cognitive decline, better cognitive speed in older adults, and greater preservation of the white matter in frontal parts of the brain (14, 15). Healthy diet with high intake of fruits and vegetables, fish, essential fatty acids and vitamin D, is beneficial for cortical sparing during normal, healthy aging (16–18). Other studies have found links between tyrosine-rich diets and cognitive functioning (19). Social and intellectual stimulation, particularly in middle age, appears to be important for maintaining cognitive abilities in old age (20, 21). Refraining from alcohol (22) and smoking (23) also contributes to maintain a healthy brain. In a similar vein, lifestyle factors such as engaging in physical activity, eating a healthy diet, restraining from substance use, and experiencing positive social interactions, have been associated with reduced risk of mental health disorders such as depression (24, 25). Other modifiable factors influencing cognitive health include body weight and cholesterol levels, sleep (26, 27), high blood pressure, diabetes, hearing loss, poor vision, and educational level (25, 28). Although much of our knowledge about relationships between brain health and lifestyle factors comes from observational epidemiological studies, and causality cannot be inferred from such associations, limited findings from multi-domain interventional studies suggest that change of lifestyle may positively affect various aspects of brain health (29).

### Rationale for the Survey

Taking care of the brain in the early stages of life is preferable as early and mid-life factors influence late-life cognitive and mental health (30) and many brain diseases are not treatable or curable. For instance, despite efforts to cure Alzheimer's disease so far not yielding the desired success, several avenues related to lifestyle factors during mid-life have been identified to potentially delay disease manifestation (28, 31). Brain health relies not only on genetic disposition but also on people's lifestyle choices. Thus, it is important to explore people's perceptions and understanding of brain health, their knowledge about measures promoting brain health as well as their willingness to choose a beneficial lifestyle to maintain a healthy brain (32, 33).

Surveys from the United States and Australia report varying levels of interest in brain health (Table 1). In 2006, the ASA-MetLife Foundation Attitudes and Awareness of Brain Health Poll was conducted among a sample of 1,000 Americans aged 42+ years. The results concluded that, although most respondents had some awareness of factors influencing brain health and believed that cognitive abilities should be checked routinely, they gave low priority to brain health compared to other health issues and did not discuss their brain health with their practitioner (36). Similarly, results from the 2013 Michael J. Fox foundation survey showed that only one in five respondents reported caring about their brain health and that most respondents underestimated the prevalence of brain disorders (37). Between 2015 and 2018, the American Association of Retired Persons (38) conducted a series of surveys in North America to explore what people do to maintain a healthy brain, with each survey focusing on one specific lifestyle factor influencing brain health (39–44). The surveys showed that the respondents often did not engage in activities that are beneficial for the brain, attributing this to factors such as lacking time, motivation, or knowledge. Moreover, many wished for more evidence regarding potential benefits. For instance, respondents to the 2017 survey on cognitive activity were uncertain about which mentally stimulating activities actually benefit brain health (39). Sixty percent of respondents to the 2017 survey on brain health and nutrition expressed willingness to eat healthier if they knew that it was beneficial for their brain health (44). In general, the respondents engaging in brain health-promoting activities reported positive outcomes such as self-perceived increased mental ability and high average mental well-being scores (39, 40, 43).

**Table 1:** Recent brain health surveys.

**Year**	**Survey title**	**Sample size**	**Age range**	**Country**
2006	American Society on Aging-MetLife Foundation Attitudes and Awareness of Brain Health Poll	1,000	42+	USA
2012	National survey about dementia risk reduction (34)	1,003	20–75	Australia
2013	Survey of Cognitive Health Beliefs, Behaviours, and Intentions (35)	900	20–89	Australia
2013	Michael J Fox Foundation for Parkinson's Research (MJFF) survey	2,013	18+	USA
2015	American Association of Retired Persons (AARP) survey on Brain Health	1,563	40+	USA
2015	Reader's Digest and the Alzheimer's Association Brain Health Survey	1,600		USA
2016	AARP Social Engagement and Brain Health Survey	2,585	40+	USA
2016	AARP Physical Activity and Brain Health Survey	1,530	40+	USA
2017	AARP Sleep and Brain Health survey	2,464	40+	USA
2017	AARP Cognitive Activity and Brain Health Survey	1,140	40+	USA
2017	AARP Brain Health and Nutrition Survey	2,033	40+	USA
2018	AARP Brain Health and Mental Well-Being Survey	2,287	18+	USA
2018	University of Michigan National Poll on Healthy Aging	1,028	50–64	USA
2019	AARP Brain Health and Dietary Supplements Survey	2,292	18+	USA
2019	MijnBreincoach survey on the association between lifestyle and brain health	590	40+	Netherlands

Results from other surveys confirm that the general public has limited awareness regarding behaviors positively affecting brain health. Although a large majority (88%) of respondents to the 2015 survey from Reader's Digest and the Alzheimer's Association, in line with current scientific evidence, believed that physical activity may decrease risk of cognitive impairment, fewer (62%) were aware that quitting smoking would be beneficial for the brain and only 5 % purposefully ate healthily to maintain brain health (45). Interestingly, according to findings from the 2018 University of Michigan National Poll on Healthy Aging (46), more than two in five adults aged 50–64 years worry about developing dementia and nearly half believe that they are at risk of developing dementia. These respondents engaged in activities such as doing crosswords or taking vitamins or dietary supplements to maintain or improve their memory, despite limited scientific evidence of potential benefits (47, 48). Similarly, a recent national survey of Australians aged 20–75 years showed that the respondents believed mental stimulation to be more efficient in reducing the risk of developing dementia than physical activity and healthy eating (34).

These surveys are informative but give no insight into people's views on brain health outside North America and Australia. Other surveys conducted in Europe explored people's willingness to change lifestyle to improve health (49) but, with few exceptions (50), do not specifically focus on brain health. The surveys also provide little information about which routines for promoting brain health people may actually implement in their daily life. Moreover, there is limited data available to assess people's willingness to learn more about their brain health, for instance by taking standardized brain health tests, and use this information to guide their lifestyle choices, although recent studies show an interest in learning more about brain health (50) and undertaking early diagnosis of dementia (51, 52).

### Objectives of Survey

The main objective of the Global Brain Health Survey is to investigate people's views on brain health globally and to further understand what may or may not motivate them to support their brain health. Specifically, we aim to explore people's:

- Perceptions of the brain and brain health,
- Interest in maintaining brain health,
- Willingness to learn more about current personal brain health state,
- Intentions and support needed to promote brain health by adopting a new lifestyle.

The survey is led by the Lifebrain consortium (9) and is executed in collaboration with national brain councils in Norway (53), Germany (54), and Belgium (55), the Brain Foundation Netherlands (56), the National University of Ostroh Academy in Ukraine (57), and the Swedish Brain Foundation (58). Importantly, the survey does not evaluate the respondents' brain health status or test their cognitive or mental abilities. Rather, it collects anonymous information about people's perceptions of, interest in and attitudes toward brain health in order to generate policy recommendations for brain health. The survey has been translated into 14 languages to reach as many people as possible across countries.

## Methods and Analysis

### Survey Design

**Figure 1:**
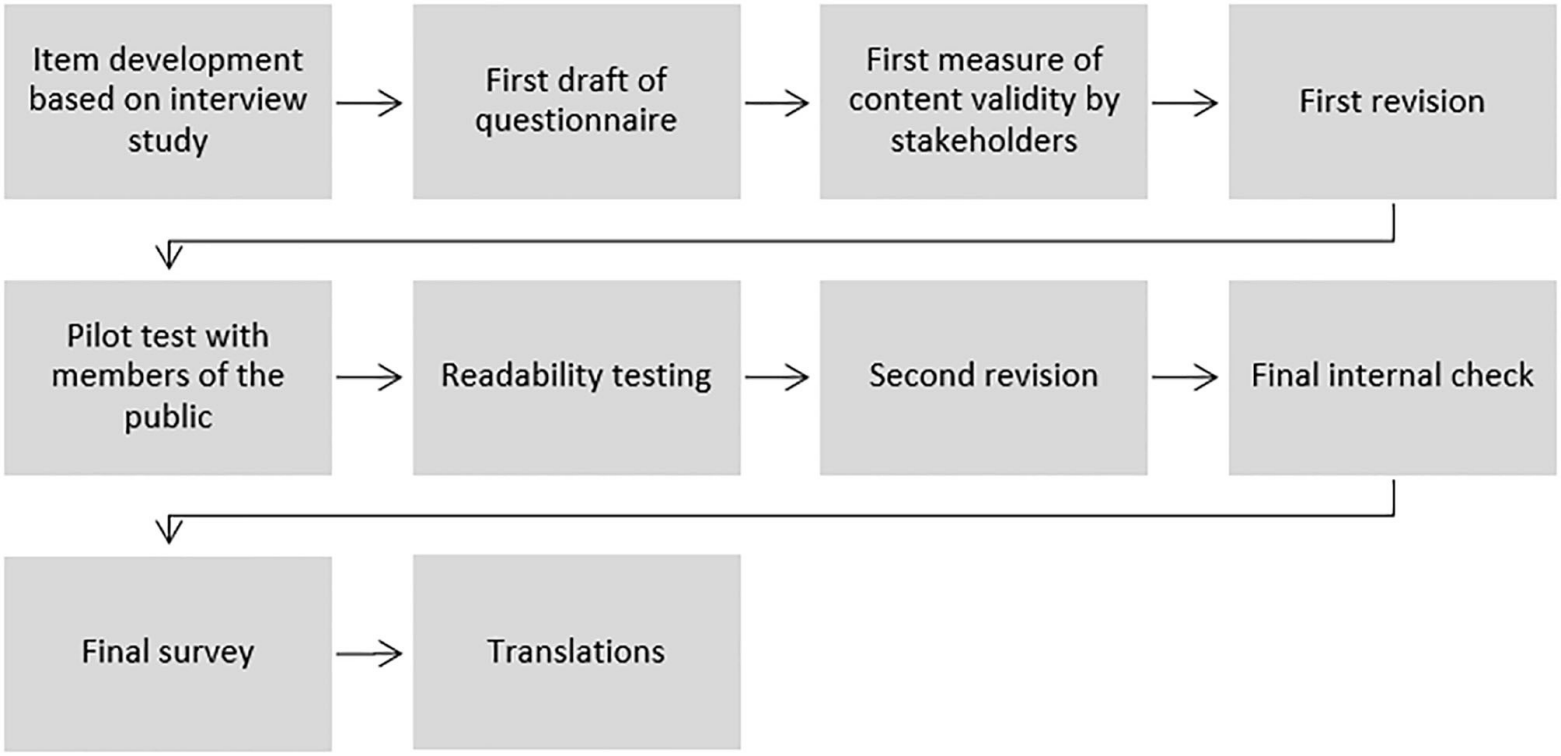
Procedure for developing the Global Brain Health Survey.

The Global Brain Health Survey is an online questionnaire of a total of 28 questions: 16 multiple-choice questions exploring the respondents' perspectives on the brain and interest in brain health, followed by 12 questions about the respondents' demographic background. All responses are anonymous. Some questions include an open text field “other” to capture information about opinions or behaviors that are not covered by the options provided. Six questions, which are related to the use of brain health tests and motivations for changing or not changing lifestyle, as well as all demographic questions, are mandatory questions although participants are free to quit the survey whenever they wish to. The survey is open to anyone interested over the age of 18 years and can be taken on a personal computer, tablet, or cell phone with internet access. The survey takes 15–20 min to complete and can be found on the Lifebrain website: https://www.lifebrain.uio.no/global-brain-health-survey/ and in Table 2. The overall procedure for developing the survey is outlined in Figure 1 and detailed below.

**Table 2:** Summary of main items of the Global Brain Health Survey.

1. How often do you think about your brain health? *(Frequently, Occasionally, Rarely, Never)*.	
2. In your opinion, to what extent do the following have an influence on brain health? *(Very strong influence, Strong influence, Moderate influence, Weak influence, No influence)*.	
• Physical health • Diet • Physical environment (e.g., air pollution, noise) • Social environment (e.g., family, social network) • Education • Profession	• Family income • Genetics and family medical history • Substance use (e.g., alcohol, smoking, drugs) • Sleeping habits • Having goals that make life meaningful
3. In your opinion, at what stages in life is it important to look after one's brain? *(Very important, Important, Moderately important, Not important)*.	
• In the womb (before birth) • Childhood (from birth to 12 years) • Adolescence (13–18 years)	• Young adulthood (19–45) • Middle age (45–65) • Old age (over 65 years)
4. Which of the following diseases/disorders do you associate with the brain?	
• Diabetes • Alzheimer's disease and other forms of dementia • Arthritis • Bipolar disorder • Cancer • Schizophrenia • Parkinson's disease	• Hypertension (high blood pressure) • Addiction (e.g., drug, alcohol) • Stroke • Depression • Migraine • Anxiety
5. How often do you engage in these activities? *(Frequently, Occasionally, Rarely, Never)*.	
• Have a healthy diet • Exercise • Sleep enough • Practice relaxing activities • Drink alcohol • Smoke • Stimulate my intellect (e.g., crosswords, learn new things)	• Strike a balance between professional and family life • Wear a helmet when cycling, skating or skiing • Take nutritional supplements such as omega 3/vitamin D • Socialize with people (e.g., friends)
6. Now, think about your brain. Which of the following activities do you do *pur*posefully for your brain health? *(Frequently, Occasionally, Rarely, Never)*.	
• Have a healthy diet • Exercise • Sleep enough • Practice relaxing activities • Strike a balance between professional and family life	• Wear a helmet when cycling, skating, or skiing • Take nutritional supplements such as omega 3/vitamin D • Socialize with people (e.g., friends)
7. You can easily take tests to check your blood pressure or cholesterol level and learn whether you are at risk of developing a heart disease. If these tests show that you are at risk, you may want to reduce your risk, for instance, by changing diet. Imagine that similar tests could be done to establish your risk of developing a brain disease. Do you think a brain health test should be: (*Select up to 3* most important criteria. You have to select at least one alternative).	
• Affordable • Quick to take • Accurate	• Painless • Subsidized by social security (via the GP) • Offered online with direct access to the results
8. Imagine a simple brain health test to learn about risk of developing a brain disease. Would you wish to take such a test? (*Select one answer)*.	
• Yes, definitely • Yes, probably	• No, probably not • No, definitely not
IF YOU ANSWERED “YES” TO QUESTION 8	
9a. Why would you take a brain health test? *Select up to two* most important reasons.	
• To get information about my cognitive and mental health • To determine my risk of developing a brain disease	• To respond if I am at risk, e.g., change my lifestyle, seek counseling, or start treatment • To prepare myself for the future (e.g., inform my family about the risk)
9b. Would you take a test even if it provides information about a disease that cannot be prevented or treated? *Select one answer*.	
• Yes, definitely • Yes, probably	• No, probably not • No, definitely not
IF YOU ANSWERED “NO” TO QUESTION 8	
9c. Why would you NOT take a brain health test? *Select up to two* most important reasons.	
• I do not want to worry about something that may not happen • I do not want to know about a disease that could not be prevented or treated	• I would be frightened by the result • There is nothing I can do for my brain health anyway
10. Imagine you undergo a brain health test and it shows that you have a risk of developing brain disease. What would be your most likely reaction? *(Definitely yes, Fairly likely, Fairly unlikely, Definitely not)*.	
• I would seek professional help (e.g., my doctor) • I would seek advice from family and friends • I would seek information online/at the library	• I would change my lifestyle if required • I would plan for the future
11. Your doctor tells that you can reduce your risk of developing a brain disease by changing your lifestyle. How likely are you to do any of the following? Think about what you would realistically do. *(Very likely, Somewhat likely, I already do that, Somewhat unlikely, Very unlikely)*.	
• Eat more healthy • Exercise more • Improve sleeping habits • Do more relaxing activities • Stimulate my brain more (e.g., learn a new language)	• Avoid alcohol • Avoid smoking • Socialize more • Do more cultural activities
12. What would motivate you to change lifestyle to improve brain health? *Select up to 3* alternatives you consider most important. You have to select at least one option.	
• If I noticed problems with my brain health (e.g., my memory worsened) • If I had been diagnosed with a brain disorder • If the lifestyle changes were fun and enjoyable • If the lifestyle changes were affordable • If my relatives or friends developed a brain disorder	• If I received personal advice about what to do (e.g., from my doctor) • If I had the support of friends/family • If it were known that the lifestyle changes are beneficial • Nothing would motivate me
13. What would prevent you from changing your lifestyle for your brain health? *Select up to 3* alternatives you consider most important. You have to select at least one option.	
• Lack of time • Lack of motivation • Lack of information about what to do • If I had to give up activities I like • If I had to take up activities that I do not enjoy • If I had to make changes by myself/alone	• If making changes was expensive (e.g., gym membership) • If I cannot be sure that the changes help • I feel no need to do anything
14. Imagine you decide to change your lifestyle to maintain or improve your brain health. What kind of assistance would you need? *Select all those that apply*.	
• Advice from my GP or specialists, e.g., nutritionist or personal trainer • Support from family members and/or friends • Group support, e.g., a walking group	• A mobile app to help with brain and physical training • Regular monitoring to review the effect of lifestyle changes
15. The public health authorities should: *(Yes, No, Do not know)*.	
• Inform the public about brain health • Raise taxes on products that are unhealthy for the brain • Subsidize food and activities that are beneficial for the brain • Offer brain health tests free of charge to citizens	• Introduce relaxation activities and sports in schools and work places • Prevent insurance companies from accessing the results of brain health tests
16. To what extent do you trust the following sources of information on brain health? *(To a great extent, Moderately, Not at all)*.	
• My general practitioner • A brain health specialist • Online medical forums • Official websites of public health authorities • Newspapers, magazines	• Social media (Facebook, Twitter, Instagram) • TV/radio (including pod-casts) • Public researchers • Scientific journals

### Item Development (Figure 1)

The survey questionnaire was developed in two phases. In phase one, the Lifebrain consortium conducted a qualitative interview study among brain research participants (*N* = 44) in Norway, Spain, Germany and the United Kingdom, to investigate their perceptions of brain health, interest in maintaining a healthy brain, and willingness to learn about personal brain health using brain health tests (59). In phase two, using the findings obtained in phase one, the Global Brain Health Survey was developed.

### Phase One: Interview Study

The phase one study included an equal distribution of men and women, the largest group of participants (57%) being older adults age 61+ followed by young adults (29%) aged 18–40, and middle-aged adults (14%) aged 41–60 years A majority of participants (59%) had a university degree and were married or in a stable relationship (64%). About a third (36%) of the participants had been educated or employed in health care-related fields (59). The study used an interview guide including 26 open questions [Supplementary Material in (59)]. Some questions were based on items in previous surveys on brain health, whereas others were developed in collaboration with stakeholders in the Lifebrain consortium's network, such as patient organizations, brain health researchers, clinicians, and cohort participants, to suit the aims of the study. Each interview participant also completed a paper questionnaire collecting demographic information, including age, educational level, marital and employment status, and employment and/or education in health care. In 2018, the research team conducted the interviews, after which interviews were transcribed, and translated to English. The interviews were coded in an inductive way using the NVivo qualitative data analysis software (QSR International Pty Ltd., version 11, 2015). A preliminary codebook was developed in which each main coding category corresponded to a specific interview question. Then, for each question, the participants' responses were organized in codes, each code representing an alternative answer. For instance, main answers to the question “What do you purposefully do to maintain a healthy brain?” were coded as “have a healthy diet,” “exercise,” and “sleep enough.” Four researchers independently coded two to three interviews to test the preliminary codebook structure. The codes were compared and discussed before the team agreed on a final codebook structure to use to code all interviews.

### Phase Two: Online Survey

In phase two, the research group used findings of the phase one interview study to develop the Global Brain Health Survey. First, the interview guide from the phase one study served to draft an initial survey questionnaire. About half of the questions from the interview guide were reformulated as the main bodies of multiple-choice questions. The other questions in the interview guide were not used because they were specific to an interview setting. Second, the corresponding answers given during the interviews, which had been coded as described above, were listed under each multiple-choice question. Finally, one question in the interview guide about motivations for changing lifestyle to take care of the brain was split into two questions to fit a survey design and to make the distinction between what “motivates” the change and what “prevents” the change from happening.

### Outline of Core Topics and Sections of the Survey

The survey questionnaire was designed to address specific topics. The first four questions map the respondent's interest in brain health, knowledge about factors influencing brain health, knowledge about stages in life when brain health promotion may be particularly important, and knowledge about brain diseases. Questions 5 and 6 explore the respondent's engagement, purposefully or not, in activities assumed to have an impact on brain health. Questions 7–10 investigate the respondent's interest in, and motivation for, undertaking or declining brain health tests to inform about individual risk of developing a brain disease. The brain health tests are hypothetical and purposefully not specified in detail as no such tests are currently available. Rather, with these items we aim to encourage the respondent to imagine future brain health tests comparable in ease of use to tests used for checking one's blood pressure or cholesterol level, and to explore their motivation for applying such tests. Questions 11–14 explore the respondent's motivation to make lifestyle changes to improve brain health, and the type of help one would need to actually make such changes. These items are assessed irrespective of whether the respondent would consider taking a brain health test or not. Finally, questions 15 and 16 investigate the respondent's views on what measures public health authorities should incorporate to promote brain health and which sources of information on brain health one considers trustworthy. Demographic questions were added at the end of the questionnaire, including questions about gender, age, country of residence, previous participation in brain research, self-perceived cognitive and mental health status, and personal experience with brain disease.

### Evaluation by Stakeholders and Revisions

From November 2018 to February 2019, the draft questionnaire was shared with Lifebrain investigators, colleagues in the Lifebrain network, representatives of patient organizations, and clinicians and lay people volunteering to check the questionnaire for validity, readability, and adequacy in addressing the domain of interest. Comments to the questionnaire were gathered by email, discussed by the research team at several online meetings, and integrated in a second draft of the questionnaire. Main comments included shortening and simplifying the questionnaire, clarifying the survey objectives, and defining brain health. In March 2019, an amended version of the questionnaire was pilot tested at a Lifebrain public lecture at the Medical Research Council in Cambridge, which gathered ~80 members of the general public. A paper version of the questionnaire was distributed, and participants were asked to comment on the suitability and clarity of the questions. Thirty-three questionnaires with written comments about the items were collected. Main comments included clarifying some questions and providing a free text option in some questions. The draft questionnaire was also discussed at a separate meeting during the same day with volunteers participating in the advisory group of the Cambridge Centre for Ageing and Neuroscience (Cam-CAN) study (60). All comments were collected, discussed by the research team, and integrated into a revised version of the questionnaire. The research team also used an online software tool (61) to check the questionnaire for readability and most survey questions were estimated to be easily understood by 15 to 16-year olds as well. In April 2019, a revised version of the questionnaire was shared with the Lifebrain researchers for a final check, and minor adjustments were made before the questionnaire was finalized.

### Survey Translation

A main objective of the survey is to reach as many people as possible in Europe and internationally. Non-native English speaking Lifebrain researchers volunteered to translate the survey questionnaire into their native language. In addition, researchers in the Lifebrain network helped translating the survey in languages not represented in Lifebrain. Each translation was either back translated to English or reviewed by at least one native person not familiar with the original version, to check for consistency of translation. Discrepancies in translation were discussed and the questionnaires amended. In June 2019, the survey was available in nine languages including English, Danish, Spanish, French, Norwegian, Catalan, German, Swedish, and Hungarian. Between July 2019 and January 2020, the questionnaire was also translated into Ukrainian, Italian, Dutch, Chinese (simplified mandarin), and Turkish. Although the survey is not available in many of the languages spoken outside Europe, the 14 languages include native languages used in other continents such as Asia and South America.

### Survey Sampling

Since launching in 2017, the Lifebrain consortium interacts with numerous research groups and civil society organizations with an interest in brain health. Using the networks of these organizations to disseminate the survey may be an efficient way to reach many individuals. The Lifebrain consortium does not have the resources and infrastructure to conduct a full-blown random population sampling approach. Thus, a convenient sampling approach was adopted, and a preliminary objective was to achieve a sample size of ~10,000 respondents. Such a sample size, across different nations, may provide unique and valuable preliminary insight into people's views on brain health, and intentions to maintain a healthy brain. In comparison, previous brain surveys collected between 590 and 2,585 responses (see Table 1). Our sample is, however, not expected to be fully representative of the general population because the individuals in the organizations' networks are more likely to be interested in brain health than the average population. In case the sample size was not achieved, the consortium would consider using the services of market research companies to boost recruitment. At present, this option is not favored, because of the inevitably high costs associated to this strategy.

### Survey Co-organizers

To maximize survey outreach, Lifebrain invited civil society organizations, including national brain councils located in Europe, to become official co-organizers of the survey. Survey co-organizers committed to disseminating the survey questionnaire in their country and through their networks. Co-organizers have their logo included in the survey dissemination material, are offered access to the aggregate survey results that they can use in their work, and can join the data analysis process. The Norwegian Brain Council (53), the German Brain Council (54), the Belgian Brain Council (55), the Brain Foundation Netherlands (56), the National University of Ostrich Academy in Ukraine (57), the Swedish Brain Foundation (58) and the Women's Brain Project agreed to become co-organizers.

### Technical Platform

The survey questionnaire has been set up using “Nettskjema,” a survey tool developed by the University of Oslo to accommodate anonymous data collection. The questionnaire is designed using standard structure for headings, text, questions, and check boxes as offered in the application menu. The survey answers do not include personal data and the time of submission and the respondent's Internet Protocol (IP) address are not stored. Respondents wishing a submission receipt may provide their email address having submitted their answers; however, the address is not stored after the receipt is sent (62). During data collection, the survey data are stored on the Nettskjema online server, which has undergone risk assessment by the Norwegian Data Protection Services (63) and was approved for the storage of anonymous data. Once the survey is completed, the data will be moved to the Lifebrain dedicated partitions on the secure and dedicated “services for sensitive data (TSD)” server at the University of Oslo, and kept there until the end of the Lifebrain project on 31 December 2021 (64). Only Lifebrain researchers have access to the anonymous data.

### Ethics Approval

In May 2019, the Regional Committees for Medical and Health Research Ethics in Norway reviewed the survey and stated that it did not require ethical approval according to the Norwegian Health Research Act, as it is anonymous (2017/653 REK Sør-Øst B). In December 2019, the University of Oxford Medical Sciences Interdivisional Research Ethics Committee (IDREC) approved the survey for dissemination in the United Kingdom (R67364/RE001). In May 2020, the Medical Ethics Review Committee of VU University Medical Center in the Netherlands approved the study for dissemination in the country's research networks. When starting the survey, respondents must confirm that they are above 18 years of age and tick off a box to consent to the use of the data for research.

### Data Availability

Before the Lifebrain project ends, we will explore the possibility to make the survey data openly available via an open science platform, for instance https://zenodo.org/. We encourage collaborations with other research groups interested in analyzing specific parts of the dataset, for instance to explore gender differences or cross-countries variations. Necessary ethics approvals will be sought to support such collaborations.

### Planned Statistical Analyses

We plan to carry out analyses in R (65). We will employ exploratory analysis strategies, for instance clustering methods or principal component analyses, to scrutinize for patterns, trends, and unanticipated results. Although the questionnaire lacks control questions to reveal irregular responses, we will perform data quality checks prior to analysis, including exclusion of non-responders and disengaged responders.

First, we will provide a thorough and comprehensive descriptive analysis of all demographic variables; describing the distributions of the demographic variables, such as gender, age group, educational level, and country of residence by means of graphical representations such as histograms and bar charts. A comprehensive description of all remaining variables describing the distributions and patterns of covariation/correlation with other questions will also be included. Second, we will explore the presence of subgroups of respondents based on similarities in answering patterns, demographic profiles and country, for instance by applying different clustering methods (66), such as latent class analyses, principal component analyses, and exploratory factor analyses. For instance, it may be particularly useful to identify the profile of respondents who are not motivated to take care of their brain health as these may require special attention from public health authorities. Lastly, we will use similar exploratory analyses as described above to specifically explore differences in response patterns between respondents in different countries.

Additionally, we aim to combine the data from specific questions under “themes” to explore how the respondents relate to different aspects of brain health. For instance, results from question 2 (In your opinion, to what extent do the following have an influence on brain health?), question 3 (In your opinion, at what stages in life is it important to look after one's brain?), and question 4 (Which diseases do you associate with the brain) may be analyzed together to better understand the respondents' perceptions of the brain and brain health in general. The same approach may be used to explore the respondents' level of engagement in brain-friendly behaviors, their willingness to take brain health tests, their motivations (or lack of thereof) for adopting new lifestyle to improve their brain health, and the type of support they require to adopt new behaviors. Of course, other themes of interest may emerge during our exploratory analysis.

### Survey Dissemination

**Figure 2:**
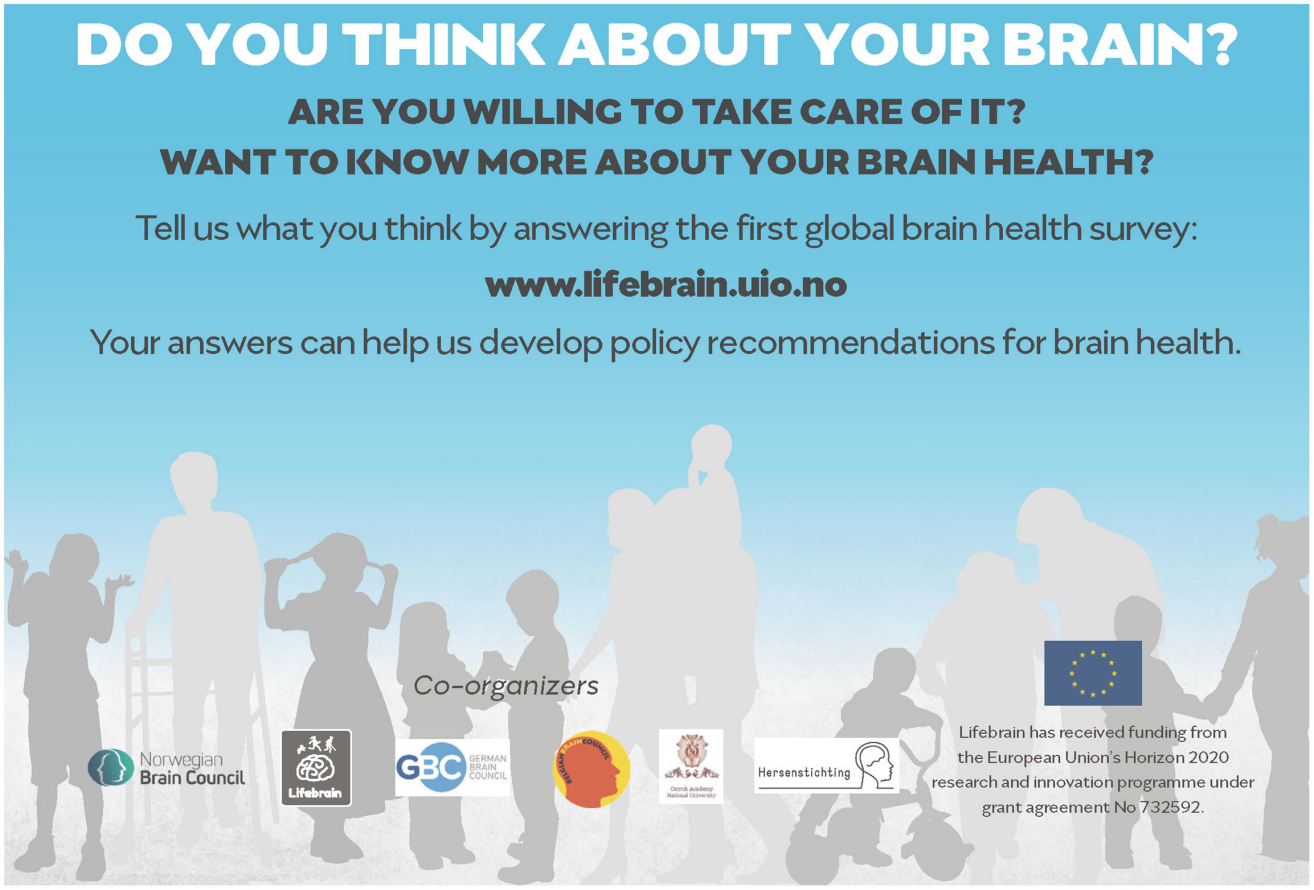
The Global Brain Health Survey postcard.

**Figure 3:**
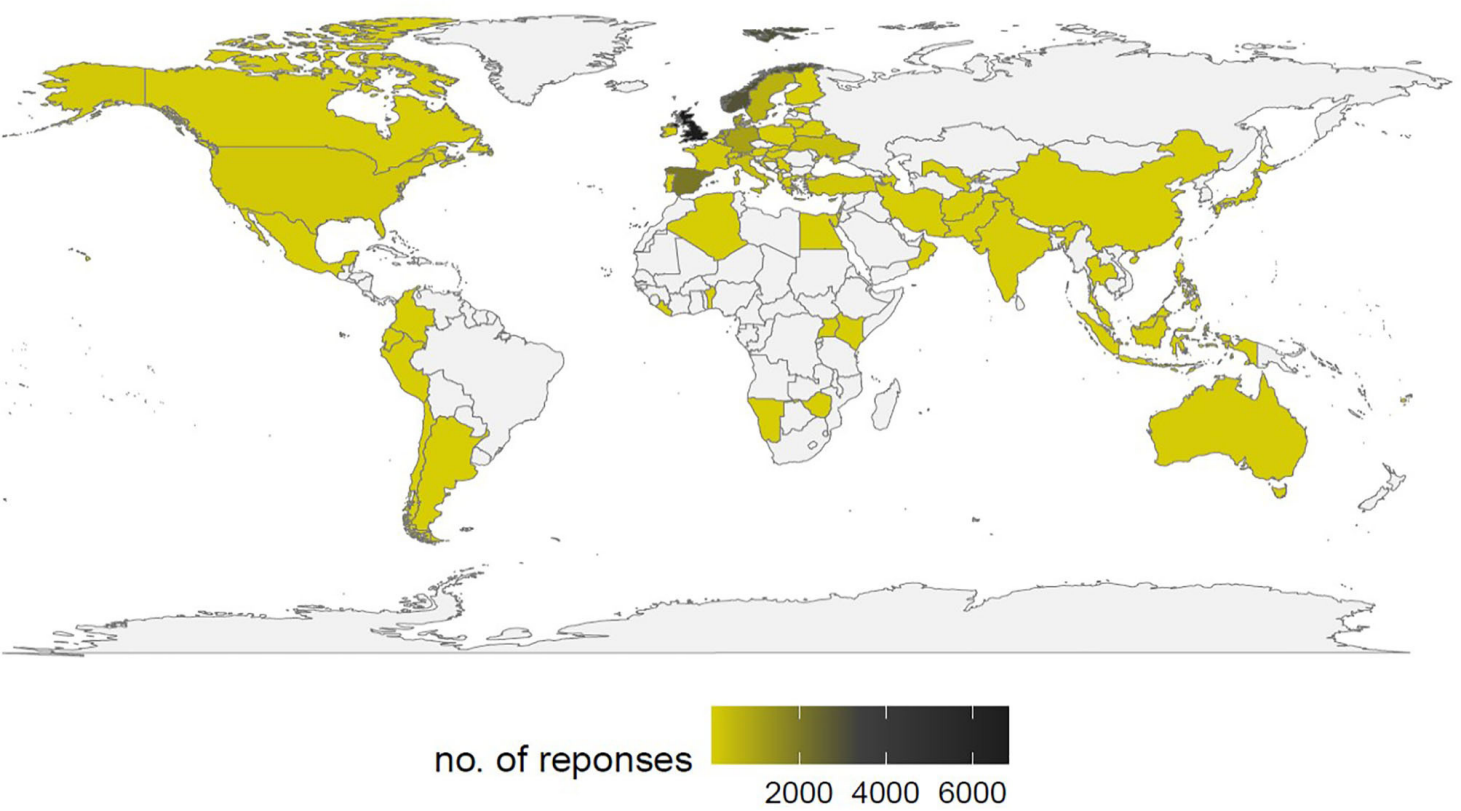
Number of survey responses by country, June 9, 2020.

The survey was officially launched on the Lifebrain website on June 4, 2019 (67) and is open until August 31, 2020. Dissemination efforts are ongoing via the networks of Lifebrain and co-organizers, including charitable organizations and departments of health, via emails, websites, newsletters, social media, post-cards (Figure 2), and posters. Dissemination channels include cohorts and registries in the Lifebrain consortium, networks of national brain councils and professional and civil societies, public conferences and lectures, and science festivals. Researchers also help distribute post-cards in public libraries, community and geriatric centers, university campuses, and public markets. Although most organizations in the Lifebrain network are in Europe, some have international outreach and disseminate the survey outside Europe. By the end of 2019, 5,730 responses were collected. As of June 9, 2020, 16,923 answers to the survey have been collected across all the languages and spanning 76 countries (Figure 3).

## Discussion

To our knowledge, this is the first multi-language online survey to investigate perceptions of brain health in the general public. The survey was developed through an extensive process of validation, revision and pilot testing. We hope that the Global Brain Health Survey will contribute to increase awareness of brain health, which still receives limited attention from policymakers despite its importance for public health.

An online questionnaire is efficient to reach out to large and diverse groups of individuals at minimum cost, as recently demonstrated by an international survey investigating public views on genetics (68). A few months after its launch, the online Global Brain Health Survey has already collected a large number of responses. However, the survey has some limitations. The interpretation of possible differences in response patterns as a function of country of residence is complicated by differences in employed dissemination strategies per country. Furthermore, although great care was taken in translating the survey into multiple languages, we cannot exclude the possibility of subtle differences in perceived meaning of individual questions. There are also concerns of response bias. First, brain health is a relatively new concept and is yet not well-defined. Although we provide the National Institute on Aging's definition of brain health (8) on the introductory page of the survey, we cannot exclude the possibility that respondents, consciously or unconsciously, maintain a slightly different conception of brain health when answering the survey. Second, some of the multiple-choice questions provide examples of lifestyle behaviors that may be beneficial for brain health. Potentially, this may bias respondents to select “socially desirable” behaviors that they would not consider otherwise. It may also be difficult to know to which extent the respondents' reported intentions to act to maintain a healthy brain are predictive of actual behavior. Several studies have shown that such intentions, also in settings where individual health risks are known, often do not materialize in practice (69) or vary, for instance, depending on educational level (70). Nevertheless, despite these possible limitations, the survey results are expected to provide useful insights into behaviors the respondents consider realistic and adoptable to maintain a healthy brain.

There is also a risk that the most resourceful groups of the general population, e.g., those who have access to e-infrastructure and know how to use it, will be the primary respondents to the survey whereas other groups like the elderly will be underrepresented. However, the proportion of elderly persons using the internet steadily increases (71). Furthermore, the survey data is collected using convenience sampling, i.e., taking advantage of the survey organizers' networks, which include cohort studies, civil and professional organizations with an interest in brain health, and lay people. Individuals participating in such networks are likely to be more interested in brain health than the general population. Also, a common experience in research on human participants is that it is easier for researchers to reach out to and recruit the resourceful and highly educated part of the population. Targeting populations with low socio-economic status and educational level may require different sampling. Finally, there is always a risk that respondents do not provide truthful responses or do not take the time to answer all questions. The survey does not include any control question to check for such scenarios. However, we expect this risk to be limited because no incentives are provided to answer the survey (72).

A central objective of the survey is to explore what people are willing to do to maintain or improve their brain health. Health policies are often implemented using a top-down approach; decision-makers translate policy recommendations on lifestyle, which are based on scientific evidence gathered by professionals, into practical measures aiming to encourage people to adopt healthy behaviors (13, 73). However, many factors may reduce people's willingness to adopt healthier lifestyles, including lack of motivation, not finding the time, lacking necessary financial resources, lack of support from family and/or health practitioners, and uncertainty regarding the actual impact of adopting healthier lifestyles on brain health (69). Typically, the general public is not involved in the design of health care policies. This survey is a bottom-up approach to policy; it aims to provide insights into what motivates people to take care of their brain, and the type of help they may need to adopt behaviors that are beneficial for brain health. The survey results will be used to develop recommendations to policymakers for the promotion of brain health in ways that are aligned with people's perceptions and daily lives. Our hope is that scientists and policy makers will evaluate scientific findings in light of these public perceptions and thereby maximize a broad acceptance and adoption of evidence-based recommendations and measures to influence the various facets of brain health. The survey results will also be published and presented at scientific conferences, shared with co-organizers, and made available to the general public in lay language and using online platforms such as the Lifebrain's and co-organizers' web pages and social media.

Finally, future studies may apply this survey to specific population groups using more targeted approaches. For instance, it might be interesting to investigate perceptions of brain health in specific contexts, such as in selected socio-economic or age groups, in schools, among teachers, students, or healthcare professionals.

## Author Contributions

IB-L led the study and drafted the manuscript. IB-L, BF, SS, CS-P, SD, CD, WB, and KM conceived the study and developed the survey questionnaire. IB-L, AM, RK, RC, BF, LN, SS, EZ, WB, KM, CS-P, PG, and AB developed the statistical analysis plan. IB-L, BF, CS-P, SD, WB, KM, EZ, PG, LN, and LZ translated the survey in their respective languages. All authors contributed to the dissemination of the survey, substantively revised the manuscript, approved the submitted version, and made substantial comments to the study design and survey questionnaire.

## Conflict of Interest

The authors declare that the research was conducted in the absence of any commercial or financial relationships that could be construed as a potential conflict of interest.
